# P-961. Preventing HIV Transmission: The Impact of Early HIV Prevention Education on The Road to Residency

**DOI:** 10.1093/ofid/ofae631.1151

**Published:** 2025-01-29

**Authors:** Jonathan E Pavia, Kyra Roa, Joseph Patrik Hornak

**Affiliations:** University of Texas Medical branch, Leauge City, Texas; University of Texas John Sealy School of Medicine, Galveston, Texas; University of Texas Medical Branch, Galveston, Texas

## Abstract

**Background:**

The emergence of HIV pre-exposure prophylaxis, or PrEP, has been effective and pivotal in reducing HIV transmission and incidence over the past decade. However, its utilization remains relatively low in the United States as barriers to acquiring PrEP persist. Some of these barriers include HIV risk perception, stigma, and provider bias. Therefore, this report investigates how increasing early career awareness of PrEP amongst fourth year medical students (MS4) matriculating into various specialties impacts their confidence around PrEP.

**Methods:**

Anonymous, online, optional, pre- and post-intervention surveys were conducted to MS4s assessing awareness, knowledge, and perceived comfort around HIV prevention and prescribing PrEP. Students received a 1.5-hour lecture regarding statistics, formulations, indications, and side effects of PrEP from an HIV specialist. Additionally, students’ knowledge around the material was gauged through three interactive patient cases regarding HIV prevention throughout lecture material.

**Results:**

Twenty-three pre- and post-intervention surveys were completed assessing familiarization and comfortability around PrEP. Seven (30%) of the students are pursuing internal medicine, five (22%) family medicine, three (13%) psychiatry, two (9%) OBGYN, and five (22%) in various specialties. Prior to attending the lecture, most students were aware of PrEP (91.3%), but most were uncomfortable initiating such a conversation with a patient (56.5%). The primary, initial limitation students cited in starting a PrEP conversation was their own knowledge around HIV prevention tools (39.1%). After the lecture, the primary constraint remained (39.1% vs. 26%), but a new challenge emerged: students’ comfort level in eliciting a comprehensive sexual history (21.7%).

**Conclusion:**

These findings demonstrate the advantages of introducing HIV PrEP education to graduating medical students. To assist in curbing HIV transmission rates, PrEP utilization must be increased across various medical specialties. Integrating PrEP education within medical school curriculum could be vital in achieving broader aims to end the HIV epidemic. Further investigation should explore alternative approaches to bolster HIV PrEP education across all levels of medical education.

**Disclosures:**

**Joseph Patrik Hornak, MD**, Innoviva Specialty Therapeutics: Advisor/Consultant|Innoviva Specialty Therapeutics: Advisor/Consultant

